# Targeting GD2-positive Refractory/Resistant Neuroblastoma and Osteosarcoma with Anti- CD3 x Anti-GD2 Bispecific Antibody Armed T cells

**DOI:** 10.21203/rs.3.rs-3570311/v1

**Published:** 2023-11-09

**Authors:** Maxim Yankelevich, Archana Thakur, Shakeel Modak, Roland Chu, Jeffrey Taub, Alissa Martin, Dana L. Schalk, Amy Schienshang, Sara Whitaker, Katie Rea, Daniel W. Lee, Qin Liu, Anthony Shields, Nai-Kong Cheung, Lawrence G. Lum

**Affiliations:** St. Christopher’s Hospital for Children, Drexel University; University of Virginia; Memorial Sloan Kettering Cancer Center; Children’s Hospital of Michigan (CHM), Wayne State University; Children’s Hospital of Michigan (CHM), Wayne State University; Children’s Hospital of Michigan (CHM), Wayne State University; University of Virginia; University of Virginia; University of Virginia; University of Virginia; University of Virginia; Wistar Institute; Karmanos Cancer Institute and Wayne State University; Memorial Sloan Kettering Cancer Center; University of Virginia

**Keywords:** Targeted T cells, bispecific antibodies, neuroblastoma, osteosarcoma, anti-CD3 x anti-GD2 bispecific antibody, hu3F8

## Abstract

**Background:**

Since treatment of neuroblastoma (NB) with anti-GD2 monoclonal antibodies provides a survival benefit in children with minimal residual disease and our preclinical study shows that anti-CD3 x anti-GD2 bispecific antibody (GD2Bi) armed T cells (GD2BATs) were highly cytotoxic to GD2+ cell lines, we conducted a phase I/II study in recurrent/refractory patients to establish safety and explore the clinical benefit of GD2BATs.

**Methods:**

The 3+3 dose escalation study (NCT02173093) phase I involved 9 evaluable patients with NB (n=5), osteosarcoma (OST) (n=3), and desmoplastic small round cell tumors (DSRCT) (n=1) with twice weekly infusions of GD2BATs at 40, 80, or 160 x 10^6^ GD2BATs/kg/infusion with daily interleukin 2 (300,000 IU/m^2^) and twice weekly granulocyte-macrophage colony stimulating factor (250 μg/m^2^). Phase II portion of the trial was conducted in patients with NB at the dose 3 level of 160 x 10^6^ GD2BATs/kg/infusion but failed to enroll the planned number of patients.

**Results:**

Nine of 12 patients in the phase I completed therapy. There were no dose limiting toxicities (DLTs). All patients developed mild and manageable cytokine release syndrome (CRS) with grade 2-3 fevers/chills, headaches, and occasional hypotension up to 72 hours after GD2BAT infusions. GD2-antibody associated pain was not significant in this study. The median OS for patients in the Phase I and limited Phase II was 18.0 and 31.2 months, respectively, whereas the combined OS was 21.1 months. There was a complete bone marrow response with overall stable disease in one of the phase I patients with NB. Ten of 12 phase II patients were evaluable for response: 1 had partial response. Three additional patients were deemed to have clinical benefit with prolonged stable disease. More than 50% of evaluable patients showed augmented immune responses to GD2+ targets after GD2BATs as measured by interferon-gamma (IFN-γ) EliSpots, Th1 cytokines, and/or chemokines.

**Conclusions:**

Our study demonstrated safety of up to 160 x 10^6^ cells/kg/infusion of GD2BATs. Combined with evidence for the development of post treatment endogenous immune responses, this data supports further investigation of GD2 BATs in larger Phase II clinical trials.

## Introduction

Despite intensive chemotherapy, autologous stem cell transplant (SCT)^1,2^, and/or anti-GD2 monoclonal antibodies therapy, many children with high-risk disease NB and OST develop recurrent disease^3,4^ which is associated with dire outcomes^1,5,6,7^. There have been no new approved drugs for OST since the 1980’s^5^.

GD2 is highly expressed on nearly all NB and 80% of OST^8,9^. Antibody-dependent cellular cytotoxicity by anti-GD2 mAbs is effective against minimal residual disease in NB^2,11,12^. We postulated that it would be feasible, safe, and effective to target NB and OST with activated autologous T- cells (ATC) armed with GD2Bi (GD2BATs). Our preclinical studies showed that GD2BATs kill GD2 + tumor targets, proliferate, and induce release of Th_1_ type cytokines upon engagement of GD2 + tumor targets^13^. In our previous clinical trials re-directing the non-MHC restricted cytotoxicity of ATC with bispecific antibody (BiAb) by engaging the TCR and tumor antigen on tumors has provided encouraging clinical and anti-tumor immune responses in breast^14,15^, prostate cancer^16,17^ pancreatic cancer^18^, non-Hodgkin’s lymphoma^19^, and multiple myeloma^20^. Infusions of BiAb armed ATC (BATs) induced endogenous responses including innate natural killer cell (NK) and cytokine/chemokine responses suggesting *in vivo* immunization of patients against their own tumor-associated antigens^14,18,21^.

In this study, we tested combination immunotherapy consisting of GD2BATs^13,22^, interleukin-2 (IL-2), and granulocyte-macrophage-colony stimulating factor (GM-CSF) in a phase I/II trial to determine safety, estimate efficacy, and evaluate immune responses. The feasibility and safety profile reported here together with the evidence of therapy-induced immune responses and observations of clinical activity support further development of the GD2BAT approach in NB.

## Material and Methods

### Production of GD2BATs

Manufacture of GD2Bi and GD2BATs was performed under BB-IND #15827 sponsored by L.G. Lum in the KCI cGMP facility, Detroit, MI and in the UV Center for Human Therapeutics, Charlottesville, VA under FDA Drug Masterfile File #19248. Clinical-grade naxitamab [humanized 3F8] was chemically heteroconjugated to clinical-grade anti-CD3 mAb (OKT3; Miltenyi, Auburn, CA) to produce GD2Bi. Lymphocytes (derived from PBMCs) were collected from the patients via apheresis, activated with 20 ng/mL of OKT3, expanded using 100 IU/mL of IL-2 and cultured at a concentration of 1 x 10^6^ mononuclear cells/mL in RPMI medium supplemented with 2% human serum and 1% L-glutamine for 14–18 days. The ATC were then harvested, armed with 50 ng of GD2Bi per 10^6^ ATC to create GD2BATs, dispersed into equal parts and cryopreserved. When ready for release for infusion, quality control assessments were performed on thawed product: included viability, proportion of CD4 + and CD8 + cells, *in vitro* cytotoxicity against KCNR GD2 + NB cell line (by mixing and incubating patients’ PBMCs with targets), testing mycoplasma, bacterial and fungal contamination, and endotoxin.

### Human Subjects.

Patients were enrolled in a Phase I/II clinical trial at CHM, UV, and MSK between November 2013 and January 31, 2022. The protocol (Clinicaltrials.gov identifier NCT02173093) was reviewed and approved by the FDA, protocol review committees, and institutional review boards at respective institutions. All guardians/patients signed informed consent before enrollment. Eligible patients were age ≤30 years at the time of enrollment and had recurrent or refractory NB, OST, or desmoplastic small round cell tumors (DSRCT). Only patients with NB were eligible for the Phase II portion. Depending on the tumor type, patients had confirmation of evaluable or measurable disease by bone marrow morphology, computed tomography, magnetic resonance imaging, and/or iodine-123 metaiodobenzylguanidine (MIBG) or positron emission tomography scans. Adequate performance score (Lansky or Karnofsky score ≥ 70) and adequate organ function (total bilirubin ≤ 1.5 times the institutional upper limit of normal (ULN), ALT ≤ 3 times the ULN, creatinine ≤ 1.5 times the ULN, and a cardiac shortening fraction ≥ 28% by echocardiogram) were required. Patients had to have an absolute neutrophil count ≥ 500/mm^3^, absolute lymphocyte count ≥ 500/mm^3^, and unsupported platelet count ≥ 50,000/mm^3^ (≥ 20,000/mm^3^ with no more than one transfusion per week for NB patients with known bone marrow involvement. A minimum of two weeks from the last chemotherapy and immunotherapy and one week from hematopoietic growth factors were required for enrollment.

Patients who had been previously treated with anti-GD2 antibodies were eligible. Date of data cutoff for this analysis was January 31, 2022.

## Clinical trial design

**Figure 1:**
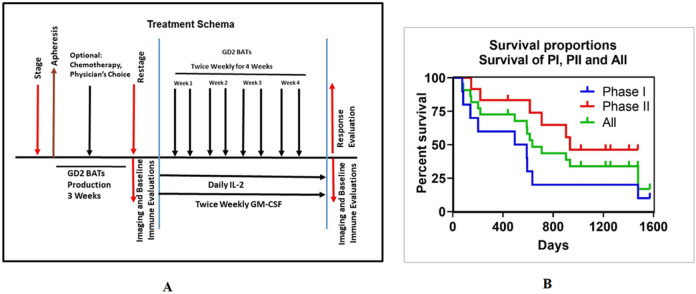
**A)** Protocol Schema. Shows the treatment schema for the Phase I/II study. **B)** Survival Curves for Phase I and Phase II. Shows the K-M Curves for Phase I (Blue, OS =18.0 months), Phase II (Red, OS=31.2 months), and All patients (Green, OS=21.0 months).

The phase I study followed a standard 3 + 3 dose escalation design with dose levels of 40, 80, and 160 x 10^6^ GD2 BATs/kg/infusion twice per week for total dose of 0.32, 0.64 or 1.28 x 10^9^ cells/kg in 8 divided doses. Low-dose IL-2 (300,000 IU/m^2^/day) and biweekly subcutaneous GM-CSF (250 μg/m^2^/dose) were administered beginning three days before the first GD2BAT infusion and ending one week after the last GD2BAT infusion (Fig. 1A)

A maximum of 18 patients were planned for the phase I study and 22 patients were needed for the Phase II cohort to confirm toxicity and estimate clinical efficacy. The primary endpoints were safety (Phase I) and ORR (PR + CR) (Phase II). Secondary endpoints included: OS, immune responses, persistence of infused cell, and early PET response after infusions. In the interval (up to 90 days) between apheresis and initiation of protocol therapy required for GD2BATs production, bridging chemotherapy with a 2-week washout was allowed. If anti-GD2 mAbs were administered during bridging, 6-week washout was required. Protocol eligibility was re-confirmed with laboratory testing, imaging and bone marrow evaluation as needed prior to commencing protocol therapy.

### Toxicity assessment.

Toxicity was assessed using Common Terminology Criteria for Adverse Event (CTCAE) v5.0 and cytokine release syndrome was graded as per the American Society for Transplantation and Cellular Therapy consensus criteria (ASTCT)^23^. Based on prior experience with BATs^14,18-21,24^, Grade 3 chills, fever, headache, nausea, emesis, and hypotension lasting < 72 hours were excluded from the definition of dose-limiting toxicities (DLTs). For this study, Grade 3 fever, nausea, or vomiting were defined as DLTs only if persisting > 72 hours. Patients were seen twice weekly, during their BAT infusion days, for evaluation of toxicities.

### Response evaluation.

Imaging and BM testing (in patients with NB) for disease evaluation was performed at enrollment, after bridging therapy (prior to protocol therapy), and 8–12 weeks after the first GD2BAT infusion. Response assessment in NB was graded using the revised International Neuroblastoma Response Criteria (INRC)^27^.

RECIST criteria were used for assessment of radiological response of measurable target lesions in non-NB tumors.

## Correlative Studies

### GD2BAT persistence studies and immune evaluations.

To assess the persistence of BATs, PBMCs from patients were stained with anti-mouse:IgG2a antibody (present on OKT3)^14^ or alternatively, anti-idiotypic antibody A1G4 specific for hu3F8 on the surface of circulating T cells at 2 and 72 hours post GD2 BAT infusion.

Patient PBMC samples were obtained prior to GD2 BAT infusion # 1 and then 1 month after the last infusion and evaluated for specific cytotoxic lymphocyte (CTL) activity using interferon-gamma (IFN-γ) enzyme-linked immunosorbent spots (ELISpots) induced by GD2-expressing NB (KCNR) and OST (MG-63) cell lines and natural killer (NK) - targeted cell line K562^13^.

Cytokines including IL-6, IL-10, IL-12, TNF-α, MIP-1β, IP-10 were quantitated by Luminex kits^21^. In selected patients with measurable soft-tissue lesions, FDG PET/CT was performed at baseline and again between infusions #5 and #8.

## Statistical Analysis.

Immune evaluations included evaluating means, standard deviations, medians, and the distributions of the data to ascertain whether normal theory methods are appropriate. Paired t-test or Wilcoxon signed-ranks test were used for comparative analyses between each pre- and post-immunotherapy (IT) time point for each patient. With a single stage Phase II trial design, it was estimated that a cohort of twenty-two Phase II patients would have 80% power at a significance level of 0.05 to detect 25% difference in response rate compared with a historical response rate of 15% or less. Due to slow recruitment, the Phase II portion of the trial only enrolled half of the planned number of patients (12 of planned 22 enrolled and 10 completed therapy) and responses after IT were analyzed descriptively without applying initially planned statistical methods in the study protocol.

## Results

### Patient characteristics.

Twelve patients (7 NB, 3 OST and 2 DSRCT) were enrolled on the Phase I study between November 2013 and December 2017, and 9 completed therapy. Twelve additional patients with NB were enrolled in the Phase II portion, and 10 completed protocol therapy. Table I summarizes the patient characteristics, diagnoses, prior therapy, product characteristics, and survival of the phase I/II patients. Reasons for not receiving therapy or completing 8 infusions in the Phase I included: failure to grow adequate ATC in 1 NB patient, rapid disease progression before GD2BATs in 1 patient with NB; and rapid disease progression leading to death after the first 3 infusions of GD2BATs in 1 patient with DSRCT. In addition, 2 patients enrolled into Phase II portion did not undergo T cell collection and did not receive therapy because of rapid disease progression after enrollment. All patients were heavily pretreated. Patients with OST and DSRCT had 2-3 and 1-2 lines of prior therapy, respectively, prior to GD2BATs. All NB patients in the Phase I portion had prior myeloablation followed by autologous stem cell transplant (SCT). Table 2 shows the distribution, disease status, prior therapy, and disease sites. Fourteen patients with NB had prior anti-GD2 antibodies.

### Preparation of cells and cell product characteristics.

Cell products were successfully manufactured in 20 of 21 patients who were apheresed. For patient IT20113, a 6-year-old male with primary refractory metastatic NB, viability of the manufactured product was only 45% (viability release ≥ 70%) and the minimum target cell number was not met (≥ 80% target dose). Cell products for the remaining 20 patients met cell expansion goals and product release criteria. Viability ranged from 73% to 96% (median 82.5%). Percentages of CD3+, CD8+, and CD4+ T cells and ^51^Cr release specific cytotoxicity against GD2+ NB cell lines are presented in Table 1. The CD8/CD4 ratios in the manufactured products ranged from 4:1 to 1:15.

### Toxicity.

In the Phase I, 9 patients received a total of 72 infusions of GD2 BATs. One patient with DSRCT quickly deteriorated due to progressive disease and therapy was discontinued after three infusions. Nine patients completed the study, each received 8 GD2 BAT infusions. No patients had their treatment stopped due to adverse effects (Table 3). All patients developed a mild, manageable form of CRS with Grades 1-3 fever, chills, headache, and nausea in 9 of 9 patients. Occasional Grade 1-2 hypotension was observed only in 3 of 9 patients after 10 of their 27 GD2 BAT infusions. Headaches and fevers lasted for up to 72 hours post infusion. The most common Grade 2 and 3 toxicities (55% each) were fever and chills, starting as early as the end of the infusion, followed by headache and hypotension. Hypotension was mild and transient and did not require vasopressors. Hypoxia was rare (1 patient had 1 episode) and did not require high-flow oxygen. Six out of 9 patients developed Grade 1-2 CRS with Grade 1 episodes after 16 of 75 infusions and Grade 2 CRS episodes after 10 of 75 infusions. Retrospective CRS grading according to ASTCT criteria did not change initial CTCAE v.5.0 grades. Most of the patients developed fever within the first 12 hours after infusion. Nausea with or without emesis and anorexia occurred in 8 of 9 patients (any Grade) and 5 of 9 patients (any Grade), respectively. Only three (33%) patients developed Grade 2 lower extremity pain that was managed with activity modifications and ibuprofen and lasted for up to three days post GD2 BAT infusion. No DLTs were observed, and the MTD was not reached. In the 10 patients treated in the limited Phase II cohort, the toxicities were similar in type and Grade to Phase I patients and there were no DLTs (Table 2). All toxicities resolved to less than Grade 3 within 72 hours. In many cases, the incidence and severity of side effects decreased with subsequent infusions.

### Clinical Responses.

#### Phase I Component

In phase I patients, observed clinical activity of GD2 BATs was limited to ungraded responses. There were no objective responses according to INRC or RECIST. Six of 9 patients who completed therapy (3/5 NB, 2/3 OST, 1/1 DSRCT) had PD, and 3 patients (2/5 NB, 1/3 OST) had SD. Six patients (one OST and 5 NB) received additional therapy after completing the study. Five of 9 patients (1 OST, 4 NB) survived 1 year post GD2BAT therapy. The median OS survival was 18.0 months for Phase I group (Fig 1B).

**Figure 2:**
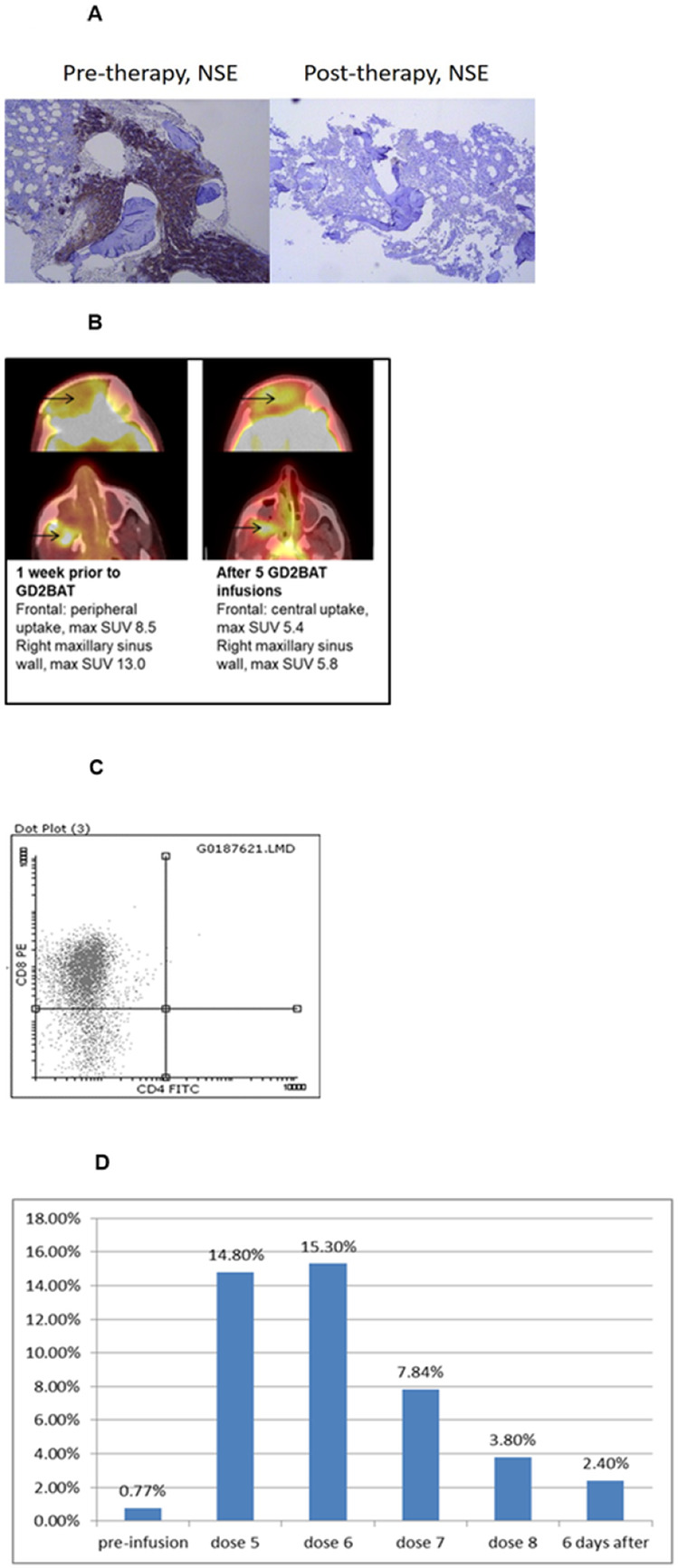
**A)** Shows a bone marrow response with clearance of neuron-specific enolase (NSE) positive tumor cells in the bone marrow of a NB patient after 8 infusions of GD2 BATs. **B)** Shows the metabolic PET changes after 5 infusions of GD2 BAT in an OST patient. **C)** Flow cytometry staining for CD4+ and CD8+ to evaluate presence of tumor infiltrating lymphocytes in the tumor for Pt # 20115. Most intranasal tumor infiltrating lymphocytes were CD8^+^. Cells were gated on CD3^+^ cells and analyzed for FITC positive CD4 and PE positive CD8 T cells. **D)** Summarizes the proportion of CD3+A1G4+ cells in the peripheral blood of patient # 8 pre-infusion and at various times during and after GD2 BATs infusions., % of CD3^+^A1G4^+^ cells shows the persistence of GD2 BATs in peripheral blood. CD3^+^A1G4^+^ cells were measured by using 2 color FACS gated on lymphocytes using anti-CD3 and A1 G4 anti-idiotypic antibody for hu3F8.

Although Phase I patients IT20111 and IT20115 did not have objective responses, their clinical courses were remarkable. Patient IT20111 had NB infiltrating his bone marrow with progressive metastatic NB bone and soft tissue disease and was treated in dose level 1. GD2BAT therapy resulted in a complete bone marrow response beginning at 1 week after therapy (Fig 2A) and stable disease in his presacral mass, retroperitoneal lymph nodes and bone metastases that lasted about 6 months. Six months after therapy, he was restarted on topotecan and cyclophosphamide chemotherapy, the same regimen he failed prior to enrolling on study. Despite progressing through this regimen previously, he had a MIBG response and clinical improvement of limping and pain and remained progression free for 2.5 additional years. He progressed 36 months after GD2BAT therapy and died of disease 47 months after therapy.

In dose level 2, Patient IT20115 with recurrent radiation-induced nasopharyngeal osteosarcoma developed an early metabolic PET response with a significant decrease in ^18^FDG PET uptake when assessed after the 6^th^ GD2BAT infusion (Fig 2B). A biopsy of the soft tissue nasal mass showed high numbers of infiltrating CD8+ cells (Fig 2C). The patient had stable disease by RECIST and was started on pazopanib after completing the study. His disease remained stable allowing for tumor resection, which extended his remission for a year. His disease progressed 14 months after GD2BAT therapy, and he died of disease 18 months later.

#### Phase II Component.

In the phase II group of 10 evaluable patients, one patient (IT00013) had PR by INRC criteria, 5 patients had SD, and 4 patients had PD at the first evaluation; however, there was additional clinical activity in some of the patients with overall SD including a patient with soft tissue mass response, and a patient with bone marrow response. The median OS for the phase II group was 31.2 months. The patient with PR was observed without additional therapies and progressed 6 months later and all 5 patients with SD were started on additional therapies.

##### Highlighted Phase II Cases.

Patient IT00013 with recurrent NB and multiple soft tissue thoracic and abdominal masses had a PR in all lesions after GD2BAT infusions with corresponding improvement in MIBG scan. He progressed 6 months after therapy, was treated with several other lines of therapy, and is free of disease 1640 days after GD2BAT therapy. Patient IT0031 with recurrent metastatic NB had PD per INRC criteria based on a new single focus of skeletal MIBG uptake, but he also had a PR in the dural intracranial soft tissue mass at the same time. He received several additional lines of therapy and is alive with disease 4 years and 2 months after GD2 BATs. Patient IT00033 with recurrent metastatic NB had SD after GD2BAT therapy with a stable MIBG scan. She had further improvement after restarting her prior salvage chemotherapy of topotecan and cyclophosphamide, followed by irinotecan and temozolomide leading to a negative MIBG scan 1 year after GD2BAT. She is alive with disease 4 years after GD2 BAT. Patient IT 000040 with recurrent metastatic NB had overall INRC SD but cleared her bone marrow disease after GD2BAT therapy. She progressed 9 months later, received additional therapies and is alive with the disease 3 years and 9 months after GD2BAT therapy.

### Immune evaluations.

#### Persistence of GD2BATs.

Staining for IgG2a (OKT3 component of the BiAb) was not interpretable due to high background staining so PBMCs were stained with anti-idiotypic antibody A1G4 specific for hu3F8 in one patient (IT00005) at MSK. Increasing numbers of circulating GD2BATs were detected above baseline (supplemental **Fig 2D**) after GD2 BAT infusions and up to 2.4% of PBMCs were GD2BATs still detected in the circulation 6 days after the last infusion.

#### Anti-GD2 Cytotoxic Activity.

**Figure 3:**
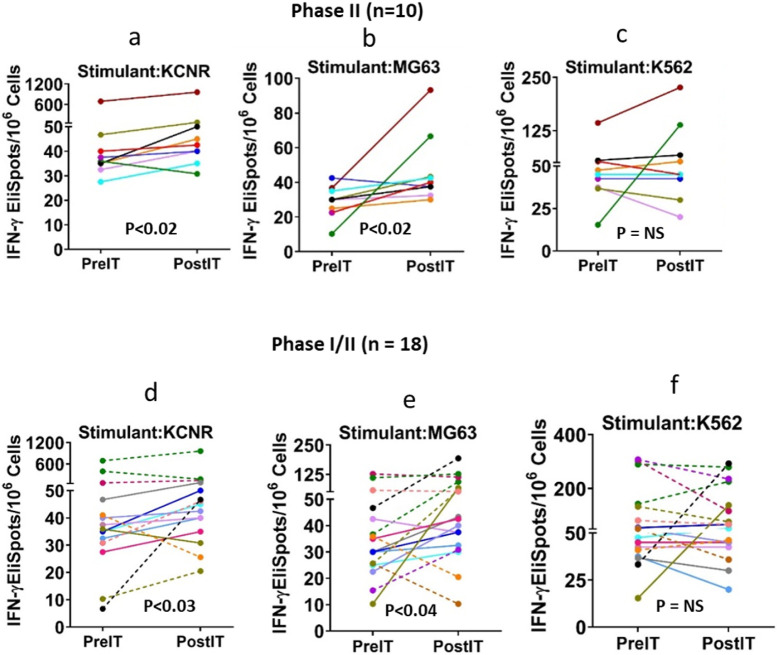
Anti-GD2 Cytotoxicity (IFN-γ EliSpots). Each panel shows the pre-IT and post-IT IFN-γ EliSpots responses from PBMC after overnight stimulation. Phase II portion: Panel a, b, and c show responses of PBMC from the **phase II** NB patients to KCNR (p<0.02), MG63 (p<0.02), and K562 (NS). Phase I/II combined: Panels d, e, and f show IFN-γ EliSpots responses of PBMC from all 18 of the phase I/II patients pre-IT and post-IT after overnight exposure to KCNR (p<0.03), MG83 (p<0.04), and K562 (Not significant, NS), respectively.

Specific anti-GD2 cytotoxicity mediated by PBMC as measured by IFN-γ EliSpots 1 month after therapy was significantly higher than prior to IT in the combined 10 phase II NB patients in responses to KCNR (Fig 3a, P<0.02), MG63 (Fig 3b, p<0.02)whereas responses to K562 were not significant. In contrast, post-IT IFN-γ EliSpots were not different for KCNR, MG63 and K562 (NK target) in the 8 phase I patients (Fig S1a-c). If IFN-γ EliSpots responses to KCNR and MG63 by PBMC of the 18 phase I and II patients were analyzed together, the post IT IFN-γ EliSpots were significantly higher than the pre-IT levels for KCNR (p<0.03) and MG63 (p<0.04). Changes in NK activity (Fig 3c and 3f) were not apparent in the phase I or II patients (Fig 3c and 3f). It is notable that Patient IT 20111 demonstrated remarkable cellular immune response that was associated with a complete BM response and overall SD. The PBMCs from the patient showed 7, 4.1, and 8.8-fold increases in IFN-γ ELISpot responses to KCNR, MG63, and K562 cell line stimulation, respectively.

#### Serum Cytokine/Chemokine Responses.

**Figure 4:**
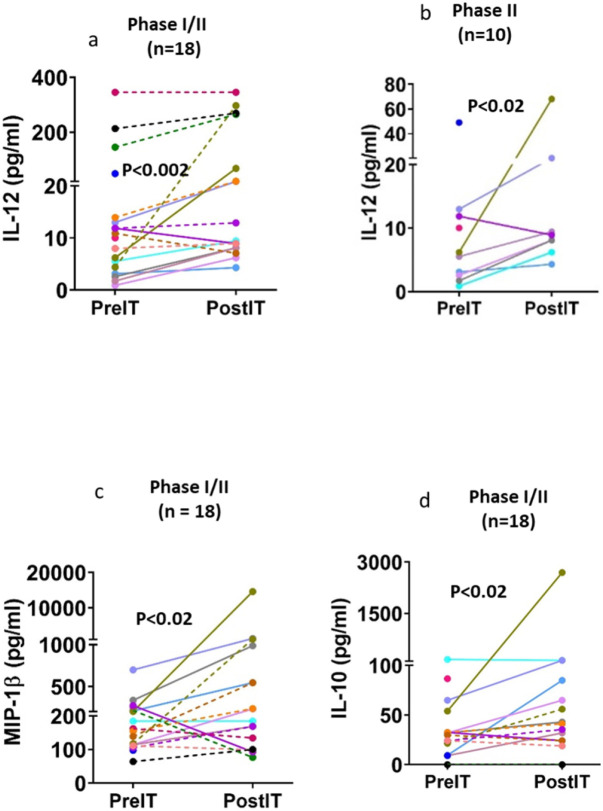
Cytokines/chemokines: Panel a (IL-12) shows significant changes between pre-IT and post-IT levels of IL-12 in all 18 Phase I/II patients (p<0.002). Panel b (IL-12) shows a significant change between pre-IT and post-IT IL-12 levels for 10 NB in the phase II patients analyzed separately (p<0.02). Panel c (MIP-1β) shows post-IT levels of MIP-1β increased significantly over pre-IT levels in 28 phase I/II patients(p<0.02). Panel d (IL-10) shows the levels of IL-10 significantly increased from pre-IT to post-IT in all 18 phase I/II patients (p<0.02). Panels e, f, and g show the pre-IT and post-IT serum levels of IL-6, TNF-α, and IP10 (all NS).

Cytokines known to be involved in immune responses and CRS were tested pre-IT and post-IT in 18 patients from combined phase I/II cohort and demonstrated significantly increased post-IT levels of the IL-12 (p <0.002) over pre-IT levels (Fig 4a) whereas the post IT levels in 10 patients of the phase II group ((Fig 4b)) had significantly increased levels over pre IT levels of IL-12 (p<0.02). Post IT serum levels of MIP1β (Fig 4c)) and IL-10 (Fig 4d) from 18 Phase I and II patients were significantly higher than pre-IT (p<0.02, and p<0.02, respectively). It is noteworthy that post-IT levels of IL-6(Fig 4**e**), TNFα (Fig 4**f**), and IP-10 (Fig 4**g**) in 18 Phase I/II patients were not elevated with respect to pre IT levels. There were no changes in the pre-IT or post IT results for IFN-γ EliSpot responses of PBMC to KCNR, MG63, and K562 and no significant changes in serum cytokine levels for IL-12, TNF-α, IL-10, IL-6, MIP1β, IP-10 in 8 Phase I patients (data not shown). There were no significant changes between pre-IT and post-IT levels of TNFα, MIP1β, and IP-10 in the Phase II patients (data not shown).

## Discussion

Despite the application of anti-GD2 mAbs, 10–20% of high-risk NB patients never achieve remission and 50–60% of those who get immunotherapy after SCT will relapse^3,4^. After patients with recurrent or refractory NB fail initial treatment and then chemoimmunotherapy, subsequent therapies including MIBG, kinase inhibitors^10^, anti-GD2 antibodies^26^, vaccines, haploidentical SCT^28^ have not induced high response rates. Although initially promising, reported clinical trials of CAR T cells targeting GD2 + failed to demonstrate formal objective responses^29,30^, except for the recent work reported from Italian group that demonstrated 63% ORR^31^. OST is responsible for 8.9% of all cancer related deaths in the pediatric age group^32^, however, there have been no new drugs for OST for more than three decades^33^. The combined progression free survival (PFS) from seven Phase II trials in OST was 12% at four months in patients with measurable disease and there were less than 5% objective responses in multiple Phase II trials over the past two decades^34^.

There were no DLTs in over 180 patients treated with BATs targeting HER2+, EGFR+, CD20+, in our Phase I/II trials of BAT therapy in metastatic breast cancer^14,15^, metastatic hormone refractory prostate cancer^16,17^, and metastatic pancreatic cancer^14,18-21^. Based on these encouraging results, we showed here that the platform could also be safely applied to target GD2 + tumors.

It was feasible to collect PBMCs by apheresis, expand the T cells, harvest, and arm ATC with GD2Bi, aliquot and cryopreserve multiple doses, and infuse the GD2BATs in pediatric outpatient clinics at MSK, CHM, and UV. Only one patient who received sirolimus as part of his previous treatment regimen failed to produce the required numbers of cells secondary to poor growth and viability. GD2BAT therapy was well tolerated in this highly pretreated pediatric patient population. The bone pain commonly associated with anti-GD2 mAb infusions was not seen except for mild leg pain in 2 patients that did not require narcotics. The MTD was not reached. Infusions were associated with mild CRS-associated symptoms with the most frequent side effects being chills, fever, headache, fatigue, and hypotension, with no ≥ Grade 3 toxicities persisting beyond 72 hours. Since the study was conducted prior to the publication of the ASTCT definition for CRS, scoring was reported using CTCAE v5.0 and were also retrospectively graded according to the ASTCT criteria^23^ and there was no difference for low grades CRS between CTCAE v.5.0 and ASTCT grading systems. All cases of CRS were CTCAE/ASTCT Grade 1 or Grade 2 at most with fever and hypotension that responded to fluids.

Although there were no objective radiological responses in the phase I portion, there was clinical activity in 2 patients (IT20111 and IT20115) with NB and OST who had a complete BM response with otherwise stable disease (IT20111) or stable tumor with decrease in FDG PET uptake (IT20115), followed by prolonged OS with additional therapies in both cases. The IT20111 patient’s clinical response was also associated with elevated anti-NB CTL, NK activity (K562 stimulated PBMCs EliSpots), and Th_1_ cytokine/chemokine levels. In the limited phase II portion, clinical activity was seen in several patients, and immunologic sensitization may have positively affected responses to subsequent therapies. For example, patient IT00013, who was chemorefractory and had multiple soft tissue, thoracic, and abdominal masses developed a radiological PR after GD2BAT therapy, progressed 6 months later, then received several additional lines of therapy resulting in a disease-free remission surviving 1640 days after GD2 BAT. Patient IT00031 had a mixed response to GD2BAT therapy, developing a PR in an intracranial mass but increased MIBG uptake in a skeletal metastasis, and he is alive with disease 4 years and 2 months later having received additional chemotherapy. Patient IT00033 had stable disease by MIBG after GD2BAT therapy, which subsequently responded to irinotecan and temozolomide, to which she was previously refractory. She is alive with the disease 4 years after GD2BAT therapy. Finally, patient IT00084 developed a bone marrow CR associated with overall SD after GD2BAT therapy, progressed 9 months later, but is alive with disease 3 years and 9 months after immunotherapy. These observations may support the concept that GD2BAT infusions “immunosensitize” the tumor to subsequent chemotherapy. A similar immunosensitization response was seen in a patient with metastatic pancreatic cancer whose peritoneal lesion progressed after three anti-CD3 x anti-EGFR BiAb armed ATC (EGFRBATs) infusions but developed a CR 2 months after restarting capecitabin that lasted 53 months^14,18^.

Specific cellular anti-GD2 CTLs developed in more than half of the patients. Anti-GD2 endogenous CTL activity was elevated in both phase I and II patients. We conclude based on our clinical study that multiple BAT infusions of up to 1.6 X 10^9^ BATs/kg in children with NB induce endogenous specific anti-tumor CTL as was also previously seen in breast, prostate, and pancreatic cancer patients;^14,15,17^ and may “sensitize” tumors so that the same prior chemotherapy becomes effective when it is restarted. This latter observation is supported by patients who became responsive after immunotherapy to checkpoint inhibitors to chemotherapy regimens to which they were previously refractory^35^.

This Phase I/II study shows IT with GD2BATs is safe and feasible in resistant/refractory heavily pretreated patients who have failed previous therapy including GD2 mAb therapies. The small extension cohort of 10 patients in the phase II portion not only confirmed safety but also showed a median OS of 31 months which is nearly double the median OS of 16.1 months reported for 469 comparable patients with recurrent NB in multiple trials meta-analysis^6^. Our results provide a strong rationale for conducting a Phase II trial that will rigorously assess the clinical benefits of GD2BATs in patients with refractory/resistant NB.

## Figures and Tables

**Table 1.** Diagnosis, Prior Therapy, Product Characteristics, and Survival

		Age (Years)	Diagnosis	Sex	Prior Therapies	Total Dose Level/ Dose x 10^9^/kg	Weight	Total Dose (x10^9^)	TX completed	Product			
										Viability (%)	Cytotoxicity (%)	CD3 (%)	CD (%)
1P1	20107	17	OST	M	CT: MAP, HD Ifosfamide	0.32	60.0	19.04	Yes	73	25	92	57
2P1	20108	10	OST	M	CT: MAP, IE	0.32	55.0	17.76	Yes	75	10	88	57
3P1	20111	15	NB	M	CT: SCT, Topo/ Cy, RT	0.32	62.9	20.08	Yes	79	53	98	31
4P1	20113	6	NB	M	CT German NB 2004, SCT, multiple lines, sirolimus	0.64	19.4	NA	No	45	NA	NA	NA
5P1	20115	26	OST	M	Multiple lines CT (MAP, IE, gemcitabine, docetaxel), Rt	0.64	66.9	42.8	Yes	74	19	99	49
6P1	20116	25	DSRCT	M	CT, pazopanib	0.64	67.4	43.1	Yes	92	33	98	64
7P1	20119	7	NB	F	ANBL12P1, SCT, vaccine, m3F8, irino/TMZ	0.64	29.3	18.75	Yes	77	3	96	6
8P1	IT00010	5	NB	M	ANBL12P1, SCT, hu3F8, RT, vaccine	1.28	18.0	23.04	Yes	91	31	95	84
9P1	IT20122	7	NB	F	ANBL0532, SCT, RT, Ch14.18	1.28	25.0	32	Yes	86	21	83	31
10P1	IT00003	31	DSRCT	M	CT, Surg, RT	1.28	91.7	37.8	No	95.8	5	94	43
11P1	IT00005	7	NB	M	ANBL12P1, MSK13-260(m3F8), CT, MSK12-230 (Hu3F8), RT,	1.28	23.3	29.9	Yes	93.6	10	98	43
12	IT00013	13	NB	M	ANBL0532, SCT, RT, Ch14.18, CT	1.28	51.4	88.96	Yes	88.6	47.9	97	52
13	IT00017	15	NB	M	ANBL12P1, SCT, RT, ANBL 0032 (ch14.18), CT, Hu3F8	1.28	75.8	78.40	Yes	93.8	17.42	94	50
14	IT00019	12	NB	M	CT, Hu3F8, RT, SCT + Haplo, NK cells	1.28	28.5	34.24	Yes	92.1	21.18	84	48
15	IT00022	10	NB	F	German NB 2004, MIBG, Hu3F8, Multiple CT	1.28	37.6	48.00	Yes	88.4	68.16	87	48
16	IT00023	11	NB	F	ANBL12P1, Ch14.18, MIBG, Hu3F8, vaccine, Hu3F8, Donor NK	1.28	25.2	25.60	Yes	83	45.7	97	45
17	IT00028	15	NB	F	CT, RT, Hu3F8, Vaccine, MIBG,	1.28	32.6	41.60	Yes	94.1	47.95	83	46
18	IT00031	19	NB	M	ABNL0532, tandem SCT, RT, Ch14.18, irino/TMZ/Ch14.18	1.28	56.6	59.04	Yes	96.9	24.88	90	63
19	IT00033	14	NB	F	ANBL12P1, SCT, RT, ANBL0032, Ch14.18; topo/cy	1.28	39.2	50.16	Yes	86.5	35.14	96	86
20	IT00040	6	NB	M	ANBL0532, tandem SCT, RT, Ch14.18	1.28	22.0	28.00	Yes	94.1	43.14	98	68
23	IT00084	25	NB	F	ANBL12P1, SCT, RT, ANBL1221 (irino/TMZ, Ch14.18)	1.28	67.2	69.04	Yes	84.2	12.11	98	40
Mean		12.5						**Mean**		83.3	29.1	94	51
Range		8-15						**Range**		73-97	3-68	83-100	20-86

CT: chemotherapy; P1: phase I; OST: Osteosarcoma; DSRCT: Desmoplastic small round cell tumor; NB: Neuroblastoma; MAP – methotrexate, doxorubicin, cisplatin, IE – ifosfamide and etoposide; topo/cy – topotecan and cyclophosphamide; irino/TMZ – irinotecan and temozolomide; RT: radiation therapy; SCT: autologous stem cell transplant; ch14.18 – chimeric GD2 mAb, m3F8 – murine 3F8 GD2 mAb, hu3F8 – humanized 3F8 GD2 mAb; PD: Progressive disease; AWD: alive with disease; DOD: Died of Disease; ANBL12P1, ANBL0532 – Children’s Oncology Group (COG) NB protocols for primary disease; ANBL1221 – COG NB protocol for recurrent /refractory disease; * for NB – primary therapy with COG ANBL protocols including chemotherapy, RT, SCT and ch14.18 (as a part of ANBL0032 or 0931) is considered as a first single line of therapy; any therapy given after the first recurrence or diagnosis of refractory disease is considered as a second line of therapy, etc.; ch14.18 was always given with IL2 and GM-CSF; ** after disease progression at 6 months and then receiving several additional lines of therapy.

**Table 2:** Distribution of Patients, Disease Status, Types of Prior Therapies, and Disease Involvement*-

	Phase I	Phase II
Male-to-female ratio	7-2	5-5
Age, median (range) at enrollment, y	12.5 (5-30)	13.5 (6-25)
Diagnosis		
Neuroblastoma	5	10
Osteosarcoma	3	
DSRCT	1	
Neuroblastoma Disease status		
Recurrent	3	7
Primary refractory	2	3
Prior therapies Neuroblastoma		
Autologous stem cell transplant	5	6
External beam radiotherapy	5	7
^131^I-MIBG	2	3
Anti-GD2 mAb	4	10
Other Phase I	3	
Prior therapies osteosarcoma and DSRCT (n=5)		
Median number of prior chemotherapy regimens	3	
Disease involvement at study entry, neuroblastoma, n	7	10
Bone marrow	3	3
Skeletal MIBG avid lesions	7	8
Measurable disease (soft tissue, lymph nodes)	4	3
Disease involvement at study entry, osteosarcoma (n=3), DSRCT (n=2)		
Bones		
Lungs	2	
Liver	2	
Abdomen	1	

* Three Phase I and 2 Phase II patients who were not treated (4) or did not complete therapy (1) were not reported in Table 2.

**Table 3:** Incidence and Grade of Toxicities by Dose Level

			Total # of Episodes by Grade			
Dose Level	Term	# patients experiencing	1	2	3	4
**Level 1**	Nausea	3	6	1		
**(n=3)**	Diarrhea	2	2			
	Chills	1	1	6		
	Fatigue	2			2	
	Headache	2	4	4	3	
	Fever	1		2	3	
	Hypotension	1		1		
	Insomnia	1		1		
	Anorexia	2	1	1	1	
	Pain	2	1	1	1	
**TOTALS**			15	17	10	
**Level 2**						
**(n=3)**	Nausea	3	3	4	2	
	Anorexia	3	6	3	3	
	Fever	3	1	6	4	
	Emesis	1			1	
	Chills	2		3	4	
	Headache	3	2	2	9	
	Pain	3		6		
	Hypotension	1		1		
	Fatigue	3	3	2	6	
	Infection, tumor site	1		1		
**TOTALS**			8	27	19	
**Level 3**						
**(n=3)**	Nausea	2	1	1		
	Anorexia	0				
	Fever	2	6	2		
	Emesis	2		3		
	Chills	0				
	Headache	2	3	2		
	Pain	1	2			
	Hypotension	1	3	5		
	Fatigue	0				
	Anemia	1			1	
	Sore throat	1	1			
	Sinus Tachycardia	1	3			
**TOTALS**			19	13	1	
**Phase II**						
**(n=10)**	Nausea	3	2	3		
	Anorexia	0				
	Fever	4	10	4		
	Emesis	3	3	5		
	Chills	3	2	12		
	Headache	6	7	7		
	Pain	1	2			
	Hypotension	1	3	5		
	Fatigue	1	1			
	Blurred vision	1	1			
	Hypoxia	1		1		
	Sore throat	1	1			
**TOTALS**			19	13	1	

## Data Availability

Data and material will be available upon request to interested researchers.

## Abbreviations

ASTCT: American Society for Transplantation and Cellular Therapy consensus criteria
ATC: activated T cells
BATs: bispecific antibody armed ATC
BiAb: bispecific antibody CTCAE: Common Terminology Criteria for Adverse Event v5.0
BM: bone marrow
Ch14.18: dinutuximab
CHM: Children’s Hospital of Michigan
CR: complete response
CRS: cytokine release syndrome
DLTs: dose-limiting toxicities
DSRCT: desmoplastic small round cell tumors
GD2Bi: anti-CD3 x anti-GD2 bispecific antibody
GD2BATs: GD2Bi armed activated T cell
GM-CSF: granulocyte macrophage colony stimulating factor
hu3F8: humanized anti-GD2 monoclonal antibody (naxitamab)
IFN-γ: interferon-gamma
INRC: international neuroblastoma response criteria
IL-2: interleukin 2
IT: immunotherapy
KCI: Karmanos Cancer Institute
mAb: monoclonal antibody
MIBG: iodine-123 metaiodobenzylguanidine
MSK: Memorial Sloan-Kettering Cancer Center
NB: neuroblastoma
NK: natural killer cell
OS: overall survival
OKT3: anti-CD3 monoclonal antibody
OST: osteosarcoma
ORR: objective response rate
PBMCs: peripheral blood mononuclear cells
PET: position emission tomography
PR: partial response
SCT: autologous stem cell transplant
SD: stable disease
TCR: T cell receptor
UV: University of Virginia
